# Association between serum Klotho concentration and hyperlipidemia in adults: a cross-sectional study from NHANES 2007–2016

**DOI:** 10.3389/fendo.2023.1280873

**Published:** 2023-11-10

**Authors:** Shaohua Yan, Wei Luo, Li Lei, Qiuxia Zhang, Jiancheng Xiu

**Affiliations:** Department of Cardiology, Nanfang Hospital, Southern Medical University, Guangzhou, China

**Keywords:** Klotho, anti-aging protein, hyperlipidemia, adults, NHANES

## Abstract

**Objective:**

The Klotho protein is a well-documented anti-aging protein known for its diverse biological functions. Hyperlipidemia is an established independent risk factor for various chronic diseases. However, there is limited understanding of the connection between Klotho and hyperlipidemia. The aim was to assess the association between serum Klotho levels and hyperlipidemia among adults.

**Methods:**

The study included 11,618 individuals from the NHANES database from 2006 to 2017. Hyperlipidemia was diagnosed following the National Cholesterol Education Program guidelines. Serum Klotho concentration was measured by an enzyme-linked immunosorbent assay kit, and the association between Klotho and hyperlipidemia was assessed by a multivariable logistic regression model. Fitted smoothing curves and threshold-effect analysis were employed to describe nonlinear relationships.

**Results:**

In our multiple logistic regression models, serum Klotho concentration was significantly associated with hyperlipidemia after adjusting for comprehensive confounders (per SD increment odds ratio (OR): 0.91; 95% confidence interval (CI): 0.86–0.97). Compared to individuals in the lowest Klotho quartile, those in the highest quartile exhibited a substantially decreased prevalence of hyperlipidemia (OR: 0.72; 95% CI: 0.58–0.90). Using a two-segment logistic regression model, we identified a U-shaped relationship between serum Klotho concentration and hyperlipidemia, with an inflection point at 1,365.5 pg/mL. Subgroup analysis did not reveal any potential moderating effects.

**Conclusion:**

This study revealed an inverse relationship between Klotho levels and hyperlipidemia. Further investigation is warranted to explore the underlying mechanism between serum Klotho and hyperlipidemia.

## 1 Introduction

Hyperlipidemia is a prevalent pathological condition in humans, stemming from disruptions in lipid metabolism that result in elevated serum lipid levels, including triglycerides and cholesterol. Hyperlipidemia elevates the incidence of stroke, diabetes, coronary heart disease, and numerous other chronic diseases (1, 2). Since hyperlipidemia is a recognized risk factor for several cardiovascular diseases, it poses significant risks to older individuals (3). In developed countries with increasingly aging populations, the prevalence of hyperlipidemia is also growing (4). It is of great clinical significance to investigate the impact of anti-aging factors on preventing hyperlipidemia.

The Klotho protein is an anti-aging protein associated with various functions and lifespan regulation (5). Mice lacking Klotho experience a spectrum of complications, including a shortened lifespan, skin atrophy, growth retardation, and osteoporosis (5). Conversely, overexpression of Klotho in mice prolongs their lifespan and offers therapeutic benefits (6). The Klotho protein is primarily expressed in renal tubular epithelial cells and exists in three distinct forms: membranous, soluble, and secreted (7). When located on the membrane, Klotho protein is a co-receptor for fibroblast growth factor 23, facilitating phosphate elimination in the urine (8). Soluble Klotho is produced by cleaving membrane-bound Klotho and is released into the bloodstream, acting as an endocrine and paracrine factor with various biological effects (9). Previous research has demonstrated that Klotho deficiencies are associated with numerous aging-related disorders, including renal disease, arteriosclerosis, hypertension, and cancers (9–11).

Several studies have explored the relationship between the anti-aging protein Klotho and lipid metabolism. In nonhuman primates, a decrease in Klotho concentration in white adipose tissues was associated with obesity induced by high-fat consumption (12). Moreover, an inverse correlation was reported between cerebrospinal fluid Klotho levels and body mass index (BMI) (13). Other researchers have shown that Klotho expression exhibits an inverse relationship with various aspects of lipid metabolism, such as inflammation, oxidative stress, and insulin resistance (14–16). However, limited evidence exists concerning Klotho levels and the risk of hyperlipidemia. This research investigated the potential relationship between Klotho levels and hyperlipidemia in the American population, using data from the National Health and Nutrition Examination Survey (NHANES).

## 2 Materials and methods

### 2.1 Data source and study population

**Figure 1:**
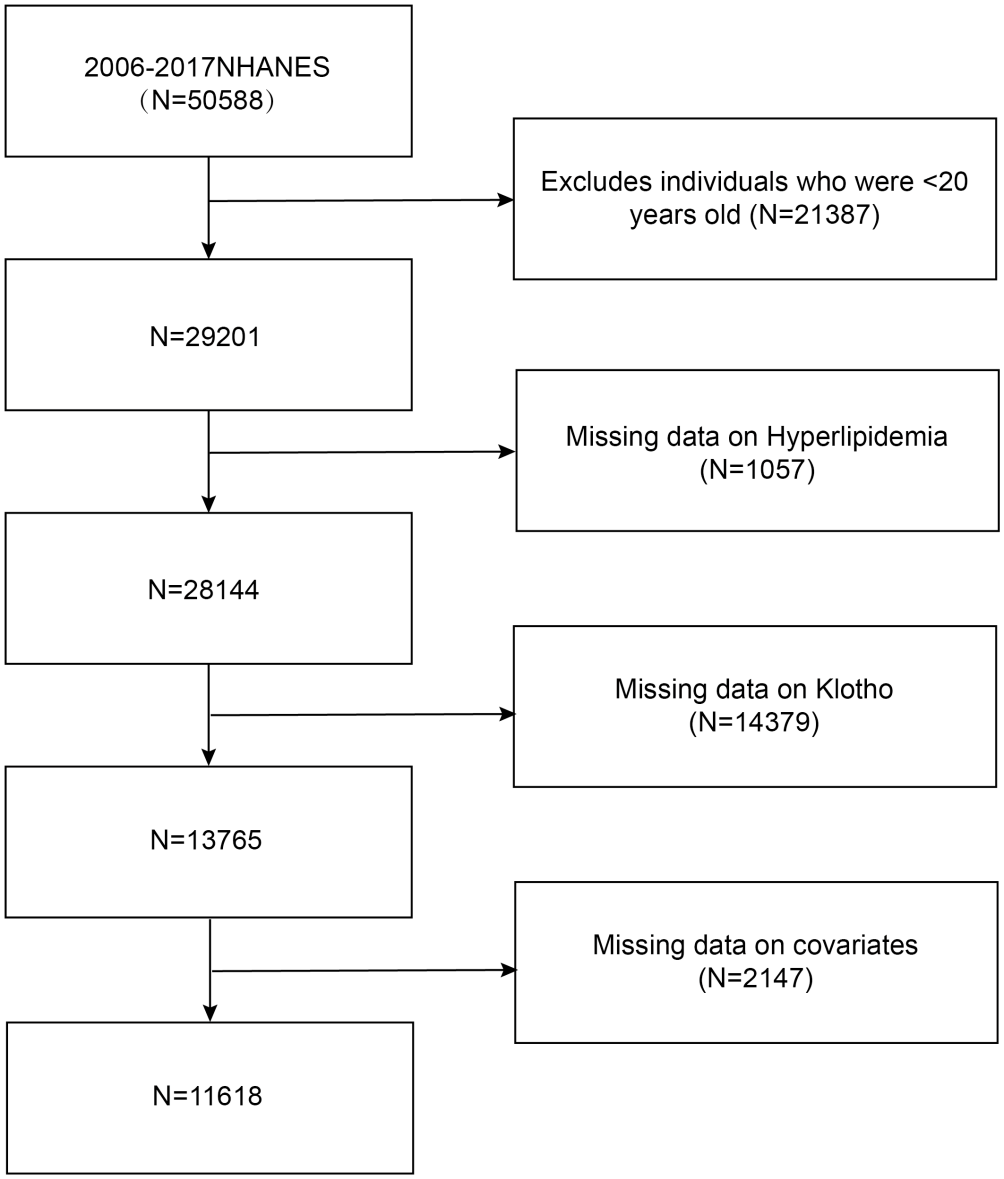
Flowchart of participant selection.

To evaluate the health condition of the noninstitutionalized American population, the NHANES played a pivotal role. NHANES is a significant survey employing a complex, multistage probability sampling methodology conducted by the National Center for Health Statistics (NCHS). The research protocol received authorization from the NCHS Ethics Review Board, and each participant provided written informed consent. A total of 29,201 adults were included in this study from the entire pool of participants. We subsequently excluded participants with missing information on hyperlipidemia (*n* = 1,057) or serum Klotho concentrations (*n* = 14,379). Participants with incomplete data on covariates were also excluded. Therefore, 11,618 individuals were ultimately recruited in our study (**Figure 1**).

### 2.2 Assessment of hyperlipidemia

The assessment of hyperlipidemia followed the guidelines established by the National Cholesterol Education Program for adults (Adult Treatment Panel III). Hyperlipidemia was defined as having total cholesterol levels of ≥200 mg/dL, triglyceride levels of ≥150 mg/dL, low-density lipoprotein levels of ≥130 mg/dL, or high-density lipoprotein levels of ≤50 mg/dL for women and ≤40 mg/dL for men (17). Alternatively, individuals who acknowledged using cholesterol-lowering medication were also classified as having hyperlipidemia (18).

### 2.3 Exposure

Participants’ blood samples were collected, preserved at −80°C, and then transferred to the Northwest Lipid Metabolism and Diabetes Research Laboratory for subsequent analysis. Klotho concentration in each participant was determined using an enzyme-linked immunosorbent assay (ELISA) kit provided by IBL International, Japan (19). To guarantee the precision of the measured data, each sample underwent duplicate testing, and the final value was calculated as the average of the two results. Further information regarding laboratory assessment can be accessed at https://wwwn.cdc.gov/Nchs/Nhanes/2007-2008/SSKL_E.htm.

### 2.4 Definition of covariates

Sociodemographic variables were available through questionnaires involving data on age, gender, race, marital status, and education level. Weight (kg) divided by height squared (m^2^) was used to determine BMI. The family poverty ratio was classified as < 1.3, 1.3–3.5, or > 3.5. Alcohol consumption was classified as never (< 12 drinks in lifetime), former (≥ 12 drinks in 1 year and no drinks in the last year, or no drinks in the previous year but ≥ 12 drinks in lifetime), and current (≥ 12 drinks and currently drinking). Smoking status was categorized as never (< 100 cigarettes in a lifetime), former (≥ 100 cigarettes but not currently smoking), and current (≥ 100 cigarettes and currently smoking). Hypertension was defined as having an average systolic blood pressure of ≥ 140 mmHg or an average diastolic blood pressure of ≥ 90 mmHg with an established diagnosis or a history of antihypertensive medications. The diagnosis of diabetes mellitus was determined by fasting glucose (mmol/L) ≥ 7.0, glycohemoglobin (%) > 6.5, the use of antidiabetic medications or insulin, or a prior diagnosis of diabetes mellitus by a physician (20). Cardiovascular disease (CVD) was classified as having angina, heart attack, congestive heart failure, coronary artery disease, or stroke.

### 2.5 Statistical analysis

NHANES analytic and reporting guidelines were followed to consider complex survey design factors in all statistical analyses (21). For continuous variables, characteristics were described as mean ± standard error (SE), while for categorical variables, they were described as proportions. Weighted one-way and weighted Chi-square analyses were employed to identify any disparities in the descriptive analyses. We utilized multivariate logistic regression models to calculate odds ratio (OR) and 95% confidence intervals (CIs) to assess the association between Klotho levels and hyperlipidemia. The crude model did not include any covariate adjustments. Model 1 was adjusted for age, gender, and race, while model 2 further included education levels, marital status, smoking status, alcohol intake, BMI, the family of poverty ratio, hypertension, diabetes, and CVD as covariates. The smoothed curve fits were generated to examine potential nonlinear relationships. A threshold effect analysis model was applied to calculate the relationship and inflection point between Klotho and hyperlipidemia. Stratified analyses were also conducted. Statistical significance was determined if the two-sided *p*-value was less than 0.05. We conducted all analyses using R Studio (version 4.2.2) and EmpowerStats (version 4.1).

## 3 Results

### 3.1 Characteristics of included participants

**Table 1** provides the baseline features of patients, categorized by Klotho quartiles. The final participants had an average age of 56.20 years, with 49.05% of them being men. The mean serum Klotho concentration was 843.67 ± 5.32 pg/mL. Individuals with increased Klotho concentrations were younger, women, better educated, less likely to have CVD and hypertension, and less likely to be drinkers and smokers.

**Table 1:** Baseline characteristics of study participants stratified by serum Klotho quartiles.

Variable	Total	Serum Klotho concentration quartiles (pg/mL)				*p-*value
		Q1 (≤ 654.33)	Q2 (654.33 – 801.40)	Q3 (801.40 – 990.93)	Q4 (> 993.93)	
Participants (*N*)	11,618	2,905	2,906	2,902	2,905	
Age (year)	56.20 ± 0.16	57.37 ± 0.23	56.39 ± 0.29	55.82 ± 0.23	55.18 ± 0.25	< 0.001
Sex *n* (%)						< 0.001
Female	5,919 (50.95)	1,387 (50.68)	1,384 (48.58)	1,517 (51.57)	1,631 (56.72)	
Male	5,699 (49.05)	1,518 (49.32)	1,522 (51.42)	1,385 (48.43)	1,274 (43.28)	
Race *n* (%)						< 0.001
Non-Hispanic White	5,282 (45.46)	1,381 (75.82)	1,416 (77.56)	1,348 (75.50)	1,137 (70.29)	
Non-Hispanic Black	2,290 (19.71)	576 (8.76)	483 (7.06)	488 (7.25)	743 (12.45)	
Mexican American	1,770 (15.23)	451 (6.06)	449 (5.90)	450 (6.42)	420 (6.29)	
Others	2,276 (19.59)	497 (9.36)	558 (9.48)	616 (10.83)	605 (10.97)	
Education level *n* (%)					
0.037
Less than high school	2,375 (20.44)	632 (13.43)	596 (12.40)	549 (11.40)	598 (13.28)	
High school	2,597 (22.35)	710 (24.52)	621 (21.47)	669 (22.64)	597 (20.43)	
College or above	6,646 (57.2)	1,563 (62.06)	1,689 (66.13)	1,684 (65.96)	1,710 (66.28)	
Marital *n* (%)						0.099
Married	7,503 (64.58)	1,881 (70.73)	1,881 (71.63)	1,899 (70.00)	1,842 (68.94)	
Separated	3,163 (27.22)	794 (22.84)	782 (20.62)	805 (23.64)	782 (22.75)	
Unmarried	952 (8.19)	230 (6.43)	243 (7.75)	198 (6.36)	281 (8.31)	
Smoking status *n* (%)						< 0.001
Former	3,470 (29.87)	943 (32.57)	903 (30.58)	845 (30.64)	779 (27.75)	
Never	5,857 (50.41)	1,310 (46.49)	1,407 (49.96)	1,517 (52.15)	1,623 (56.26)	
Current	2,291 (19.72)	652 (20.94)	596 (19.45)	540 (17.21)	503 (15.99)	
Alcohol intake *n* (%)						0.002
Former	2,528 (21.76)	663 (18.68)	594 (16.39)	618 (18.22)	653 (18.67)	
Never	1,639 (14.11)	342 (8.28)	409 (10.56)	394 (9.40)	494 (12.49)	
Current	7,451 (64.13)	1,900 (73.05)	1,903 (73.05)	1,890 (72.38)	1,758 (68.84)	
Family of poverty ratio *n* (%)						0.581
≤ 1.3	3,483 (29.98)	918 (17.89)	868 (16.80)	837 (16.53)	860 (17.67)	
1.3–3.5	4,201 (36.16)	1,059 (33.81)	1,033 (32.95)	1,040 (31.81)	1,069 (32.94)	
> 3.5	3,934 (33.86)	928 (48.30)	1,005 (50.25)	1,025 (51.66)	976 (49.38)	
BMI (kg/m^2^)	29.61 ± 0.11	29.67 ± 0.14	29.67 ± 0.17	29.63 ± 0.19	29.44 ± 0.21	0.759
CVD *n* (%)						0.003
Yes	1,612 (13.88)	501 (13.29)	397 (10.78)	377 (10.75)	337 (9.63)	
No	10,006 (86.12)	2,404 (86.71)	2,509 (89.22)	2,525 (89.25)	2,568 (90.37)	
Diabetes *n* (%)						0.278
Yes	2,796 (24.07)	731 (18.56)	645 (17.30)	663 (17.35)	757 (19.50)	
No	8,822 (75.93)	2,174 (81.44)	2,261 (82.70)	2,239 (82.65)	2,148 (80.50)	
Hypertension *n* (%)						0.009
Yes	6,296 (54.19)	1,679 (51.79)	1,575 (48.43)	1,511 (46.55)	1,531 (47.63)	
No	5,322 (45.81)	1,226 (48.21)	1,331 (51.57)	1,391 (53.45)	1,374 (52.37)	

CVD, cardiovascular diseases; DM, diabetes mellitus; BMI, body mass index; Q, quartile. All values are presented as a number and proportion (%) or mean and SE.

### 3.2 The association of serum Klotho concentration with hyperlipidemia

**Table 2** displays three multiple regression analyses assessing the association between Klotho concentration and hyperlipidemia. The association remained significant in the crude model (per standard deviation (SD) increment OR: 0.87; 95% CI: 0.82–0.93) and model 1 (per SD increment OR: 0.90; 95% CI: 0.85–0.95). In model 2, the relationship between Klotho and hyperlipidemia remained robust (per SD increment OR: 0.91; 95% CI: 0.86–0.97), indicating that each SD increment in Klotho was associated with a 9% decreased risk of hyperlipidemia. When serum Klotho was assessed in quartiles, individuals in the highest Klotho quartile had a substantially lower prevalence of hyperlipidemia than those in quartile 1 (OR: 0.72; 95% CI: 0.58–0.90).

**Table 2:** Association between Klotho and hyperlipidemia among US adults in NHANES 2007–2016.

Klotho (pg/mL)	Crude model		Model 1		Model 2	
	OR (95% CI)	*P*-value	OR (95% CI)	*P*-value	OR (95% CI)	*P*-value
Per SD increase	0.87 (0.82, 0.93)	< 0.001	0.90 (0.85, 0.95)	< 0.001	0.91 (0.86, 0.97)	0.005
Quartiles						
Q1 (≤ 654.33)	Reference		Reference		Reference	
Q2 (654.33 – 801.40)	1.00 (0.82, 1.22)	0.971	1.03 (0.84, 1.25)	0.787	1.05 (0.86, 1.28)	0.614
Q3 (801.40 – 990.93)	0.80 (0.66, 0.96)	0.019	0.82 (0.68, 1.00)	0.049	0.84 (0.69, 1.02)	0.083
Q4 (> 993.93)	0.65 (0.53, 0.80)	< 0.001	0.70 (0.57, 0.86)	0.001	0.72 (0.58, 0.90)	0.004
*p* for trend	< 0.001		< 0.001		< 0.001	
Categories						
Q1–Q3 (≤ 993.93)	Reference		Reference		Reference	
Q4 (> 993.93)	0.70 (0.60, 0.82)	< 0.001	0.74 (0.63, 0.87)	0.001	0.75 (0.63, 0.89)	0.002

Crude model: no cofounder.

Model 1: adjusted for age, gender, etc.

Model 2: adjusted for age, gender, race, education, alcohol intake, smoking status, body mass index, the family of poverty ratio, hypertension, diabetes, and CVD.

OR, odds ratio; CI, confidence interval.

### 3.3 The analyses of nonlinear relationship

**Figure 2:**
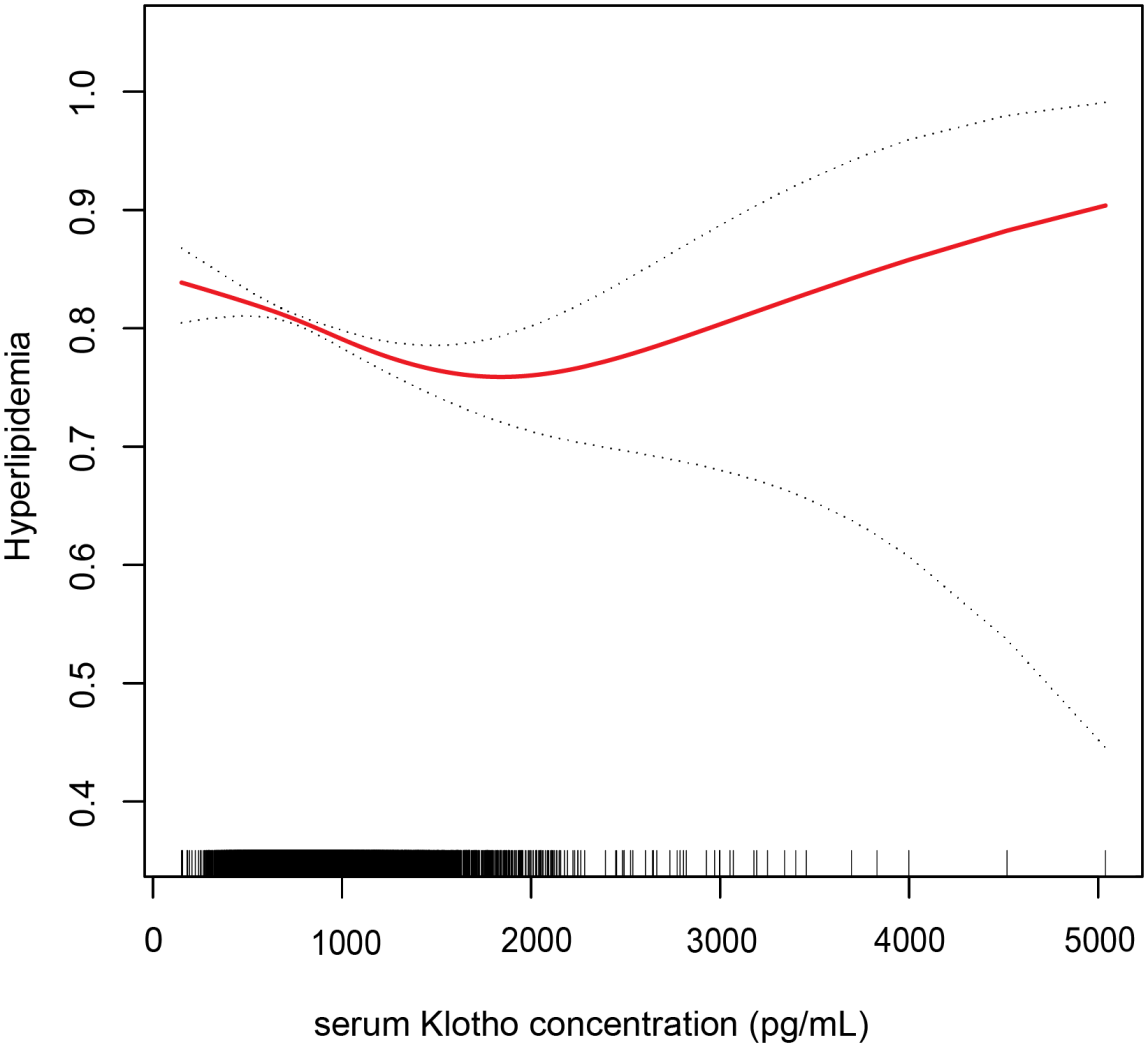
Association between serum Klotho concentration and hyperlipidemia. Adjusted for age, gender, race, education, alcohol intake, smoking status, body mass index, the family of poverty ratio, diabetes, hypertension, and CVD. The red line and dashed line indicate the odds ratio and 95% confidence interval, respectively.

We employed a smoothed curve fitting to illustrate the nonlinear relationship between Klotho levels and hyperlipidemia. Using a two-segment logistic regression model, we identified a U-shaped relationship between serum Klotho and hyperlipidemia, with a distinct inflection point at 1,365.5 pg/mL (**Figure 2**). On the left side of this inflection point, the OR (95% CI) between Klotho concentration and hyperlipidemia prevalence was 0.9996 (0.9994, 0.9998). However, no significant relationship was observed on the right side of the inflection point, with an OR of 1.0002 (0.9998, 1.0006) (**Table 3**).

**Table 3:** Threshold analyses of serum Klotho on hyperlipidemia using two-segment regression models.

Inflection point of Klotho (pg/mL)	Effect size (OR)	95% CI	*p*-value
< 1,365.5	0.9996	(0.9994, 0.9998)	< 0.0001
> 1,365.5	1.0002	(0.9998, 1.0006)	0.3633

Adjusted for age, gender, race, education, alcohol intake, smoking status, body mass index, the family of poverty ratio, hypertension, diabetes, and CVD.

### 3.4 Subgroup analysis

**Figure 3:**
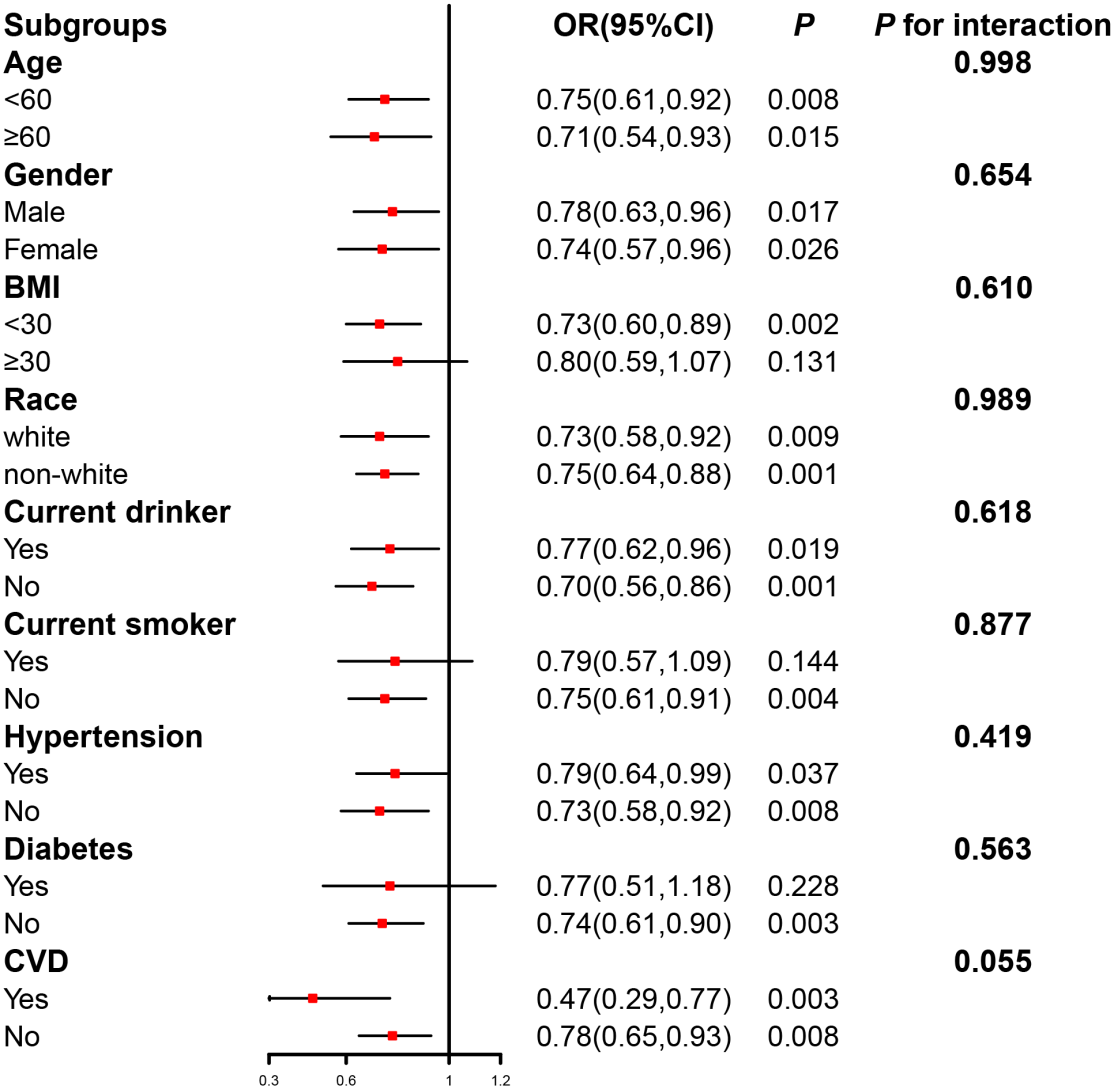
The relation of serum Klotho concentration (Q1–Q3 vs. Q4) with the prevalence of hyperlipidemia in various subgroups. Adjusted for age, gender, race, education, alcohol intake, smoking status, body mass index, the family of poverty ratio, hypertension, diabetes, and CVD, if not stratified.

To evaluate the robustness of the connection between serum Klotho and hyperlipidemia, subgroup analyses were conducted to stratify the relationship based on age (< 60/≥ 60 years), gender (man/woman), BMI (< 30/≥ 30 kg/m^2^), race (White/non-White), current drinker (yes/no), current smoker (yes/no), hypertension (yes/no), diabetes (yes/no), and CVD (yes/no). We found no significant interactions in the relationship between serum Klotho concentration (Q1–Q3 vs. Q4) and the prevalence of hyperlipidemia across various subgroups (*p* > 0.05 for the interaction) (**Figure 3**).

## 4 Discussion

The study aimed to investigate the potential relationship between serum Klotho concentration and the prevalence of hyperlipidemia. Our results indicated an inverse relationship between Klotho levels and hyperlipidemia, even after adjusting for covariates and performing subgroup analyses. Furthermore, we identified a nonlinear relationship between Klotho and hyperlipidemia.

Previous investigations have shown the potential connections between aging and lipid metabolism. The aging process has been reported to be related to increased plasma lipoproteins and reduced plasma triglyceride clearance due to diminished lipoprotein lipase activity (22). Furthermore, aging is associated with the redistribution of adipose tissue, involving the transfer of fat from subcutaneous depots to visceral depots (23). Individuals with higher levels of adiponectin, expressed in adipose tissue, are reported to have extended lifespans (24). Some interventions aimed at delaying aging, such as calorie restriction, gene mutations (such as insulin-like growth factor 1 receptor (IGF-1R)), and pharmaceuticals (such as metformin), have demonstrated the ability to impact lipid metabolism (25–27). Li et al. showed that age-related alterations in DNA methylation are associated with impaired lipid metabolism, which is associated with the aging process (28). According to Cui’s research, the anti-aging protein Klotho is adversely linked with visceral adiposity (29). A recent study has demonstrated that cerebrospinal fluid Klotho levels are inversely correlated with BMI (13). However, no previous assessments have been made regarding the impact of plasma Klotho on lipid metabolism or the potential correlation between hyperlipidemia and Klotho levels. Our study identified a U-shape relationship between serum Klotho and hyperlipidemia, with an inflection point of 1,365.5 pg/mL. Therefore, our study offers credible data for future research to uncover the underlying mechanisms of the relationship between the anti-aging protein Klotho and hyperlipidemia.

Several possible mechanisms support our findings. A study conducted by Martín-Núñez revealed that reduced levels of Klotho were associated with a proinflammatory state characterized by an elevated tumor necrosis factor-alpha/interleukin-10 ratio and increased levels of C-reactive protein (30), which could potentially impact various aspects of lipid metabolism. Moreover, Klotho expression decreased insulin production and suppressed the downstream pathway of IGF-1R (31). Furthermore, Klotho has been linked to increased resistance to oxidative stress, which results from the accumulation of reactive oxygen species and is achieved through enhanced phosphorylation of Forkhead transcription factor 3a (32). Wang’s study demonstrated that Klotho could mitigate superoxide production and oxidative damage through the cyclic adenosine monophosphate/protein kinase A pathway (33). Consequently, we hypothesized that the potential mechanism by which Klotho negatively correlates with hyperlipidemia might involve anti-inflammatory effects, insulin resistance, and antioxidants.

The present study has multiple strengths. Firstly, we utilized a national sample to analyze, enabling us to interpret the weighted outcomes as representative of the American population at the national level. Furthermore, we conducted sensitivity analyses to ensure consistent results across various subgroups. However, several limitations should be considered. Firstly, as this was a cross-sectional survey, it was challenging to establish a precise cause-and-effect relationship between hyperlipidemia and Klotho. Secondly, we could not track the duration of medication usage in patients. Furthermore, Klotho levels varied over time and displayed a circadian rhythm, indicating a potential influence of the differences in blood collection periods on the results.

## 5 Conclusion

The study showed a significant association between serum Klotho levels and hyperlipidemia among American adults. Further longitudinal research and cohort studies conducted in different regions are necessary to evaluate the cause-and-effect relationship more thoroughly.

## Data availability statement

The datasets presented in this study can be found in online repositories. The names of the repository/repositories and accession number(s) can be found below: www.cdc.gov/nchs/nhanes/.

## Ethics statement

The studies involving humans were approved by National Center for Health Statistics Ethics Review Board. The studies were conducted in accordance with the local legislation and institutional requirements. The participants provided their written informed consent to participate in this study.

## Author contributions

JX: Supervision, Writing – review & editing, Project administration. SY: Conceptualization, Formal Analysis, Writing – original draft, Software. WL: Software, Conceptualization, Formal Analysis, Writing – original draft. LL: Investigation, Validation, Resources, Writing – review & editing. QZ: Investigation, Resources, Validation, Writing – review & editing.
